# Mobile and Wireless Autofluorescence Detection Systems and Their Application for Skin Tissues

**DOI:** 10.3390/bios15080501

**Published:** 2025-08-03

**Authors:** Yizhen Wang, Yuyang Zhang, Yunfei Li, Fuhong Cai

**Affiliations:** 1 Key Laboratory of Biomedical Engineering of Hainan Province, School of Biomedical Engineering, Sanya Research Institute, Hainan University, Sanya 572000, China; 23220854090034@hainanu.edu.cn (Y.W.); 20233008301@hainanu.edu.cn (Y.Z.); 2 College of Electronic and Information Engineering, Hebei University, Baoding 071002, China

**Keywords:** skin autofluorescence, portable detection, CMOS image sensor, bluetooth communication

## Abstract

Skin autofluorescence (SAF) detection technology represents a noninvasive, convenient, and cost-effective optical detection approach. It can be employed for the differentiation of various diseases, including metabolic diseases and dermatitis, as well as for monitoring the treatment efficacy. Distinct from diffuse reflection signals, the autofluorescence signals of biological tissues are relatively weak, making them challenging to be captured by photoelectric sensors. Moreover, the absorption and scattering properties of biological tissues lead to a substantial attenuation of the autofluorescence of biological tissues, thereby worsening the signal-to-noise ratio. This has also imposed limitations on the development and application of compact-sized autofluorescence detection systems. In this study, a compact LED light source and a CMOS sensor were utilized as the excitation and detection devices for skin tissue autofluorescence, respectively, to construct a mobile and wireless skin tissue autofluorescence detection system. This system can achieve the detection of skin tissue autofluorescence with a high signal-to-noise ratio under the drive of a simple power supply and a single-chip microcontroller. The detection time is less than 0.1 s. To enhance the stability of the system, a pressure sensor was incorporated. This pressure sensor can monitor the pressure exerted by the skin on the detection system during the testing process, thereby improving the accuracy of the detection signal. The developed system features a compact structure, user-friendliness, and a favorable signal-to-noise ratio of the detection signal, holding significant application potential in future assessments of skin aging and the risk of diabetic complications.

## 1. Introduction

Skin autofluorescence (SAF) detection has significant applications in biomedicine. Currently, this technology has been applied in clinical research and risk assessment for various diseases. In the field of chronic metabolic diseases, SAF has been widely used for risk prediction and dynamic monitoring of diabetes, cardiovascular diseases, cardiorenal syndrome, etc. [1,2]. In patients with kidney disease, elevated SAF has been shown to correlate with higher risk of cardiovascular events, all-cause mortality, and progression of renal dysfunction, indicating its value as a cumulative metabolic stress biomarker [3,4]. In the area of oncological diseases, SAF is mainly utilized for the early identification and risk stratification of skin tumors such as basal cell carcinoma and melanoma [5]. Additionally, SAF is gradually being employed for the monitoring of disease conditions and therapeutic efficacy tracking in chronic inflammatory and degenerative skin diseases, such as psoriasis, atopic dermatitis, and photoaging [6]. The above studies indicate that SAF technology has good adaptability and clinical translation prospects in disease assessment under various mechanism backgrounds.

However, due to the presence of scattering and absorption in biological tissues, the detection efficiency of optical signals is relatively low, and large-scale optical systems are typically required to achieve a high signal-to-noise ratio (SNR) [7]. In current clinical and biomedical research, numerous high-performance optical instruments are widely used for imaging and analyzing biological tissues such as the brain, liver, lungs, and tumors. For example, confocal laser scanning microscopy (CLSM) captures fluorescence signals from specific focal planes via point-scanning and spatial pinhole configurations, making it well-suited for observing cellular morphology, intercellular connections, and pathological features in tissue sections or live samples [8]. Multiphoton microscopy (MPM) employs femtosecond near-infrared lasers to induce nonlinear excitation, providing deep tissue penetration and submicron resolution. It is commonly used for imaging endogenous fluorophores such as nicotinamide adenine dinucleotide plus hydrogen (NADH) and flavin adenine dinucleotide (FAD), enabling metabolic and tumor microenvironment assessments [9]. Fluorescence lifetime imaging microscopy (FLIM) adds a temporal dimension by measuring fluorescence decay times, allowing for evaluation of metabolic status and molecular interactions [10]. Other systems, such as photoacoustic microscopy, Raman spectroscopy, and optical coherence tomography (OCT), are also widely applied for vascular imaging, molecular profiling, and cross-sectional structural analysis [11,12,13]. In addition to these established techniques, broadband spatial–temporal–spectral sensing approaches—particularly those utilizing short-wave infrared (SWIR) bands—have demonstrated promising potential in biological sample characterization, providing complementary spectral and spatial information [14]. However, while these instruments offer powerful imaging and analytical capabilities, they typically rely on high-precision lasers, optical elements, and mechanical scanning systems, which makes them bulky and costly. Consequently, their use is largely restricted to laboratory or hospital settings, limiting their applicability in real-time or wearable health monitoring scenarios [15].

On the other hand, even specialized skin fluorescence detection devices require relatively long exposure times to obtain fluorescence signals with acceptable SNR. A representative example is the AGE Reader, which is a commercial medical tool designed for the noninvasive assessment of advanced glycation end-products (AGEs) accumulated in the skin. It is commonly used for evaluating the long-term risk of diabetes, cardiovascular disease, and metabolic syndrome [16,17,18]. AGEs are stable compounds that gradually accumulate in tissues under conditions of chronic hyperglycemia and oxidative stress, and they exhibit characteristic autofluorescence. The AGE Reader uses ultraviolet light to excite the volar forearm skin, collects the resulting autofluorescence emitted by AGE molecules, and quantifies the level of AGE accumulation as a proxy for long-term metabolic burden [19]. The device offers noninvasive, rapid, and easy-to-use evaluation and has been employed in diabetes screening and chronic disease management, particularly in primary care settings [20,21]. However, to ensure reliable and reproducible measurements, the AGE Reader typically requires continuous exposure for 8–12 s, during which the subject must remain in a fixed posture and under controlled ambient lighting conditions. Any fluctuations in light source intensity, body movement, or ambient light can affect signal quality. These requirements limit the use of the AGE Reader in dynamic or mobile applications [22,23,24]. Furthermore, while the device is relatively portable, it remains a desktop-based system and cannot be fully integrated into wearable or continuous monitoring platforms [25].

Overall, the deployment of skin autofluorescence (SAF) technology—whether through advanced imaging systems or clinical-grade detection devices—is currently hindered by two major technical limitations: First, the bulky and complex nature of existing hardware; and second, the dependence on long exposure times and stable environmental conditions to ensure signal fidelity. These factors restrict the translation of SAF from controlled laboratory environments to real-world, wearable, or continuous health monitoring applications. Although SAF holds considerable promise as a noninvasive, physiologically relevant biomarker for chronic disease evaluation, its broader adoption requires significant advancements in system miniaturization, signal acquisition efficiency, and environmental robustness [26].

In 2024, Samsung Electronics introduced the “AGEs Index” feature in its Galaxy Watch7 and Galaxy Watch Ultra smartwatches, attempting to noninvasively estimate skin AGEs levels through its BioActive sensor module [27]. Although this feature remains in the trial phase and is currently intended for health trend tracking rather than clinical diagnostics, its integration strategy and product form offer a demonstrative example of SAF implementation in mobile devices [28]. This marks a significant step toward the miniaturization and continuous application of the technology, as also demonstrated in recent studies proposing portable SAF systems for biological sample analysis [29]. With further improvements in signal stability and acquisition efficiency, SAF may become more applicable to portable platforms and clinical screening scenarios [30].

However, in the field of optical detection for biological tissues, individual variability during experimental procedures poses a significant constraint on the development of this technology. For instance, differences in skin pigmentation among individuals can interfere with the detection of scattered or fluorescent light [31]. When the detection accuracy of physiological parameters is low, it further undermines the clinical effectiveness of the detection method. Moreover, in contact-based optical detection systems for biological tissues—such as blood oxygen monitoring in smartwatches—the pressure exerted on the skin can alter the distribution of subcutaneous blood, causing changes in local absorption coefficients and consequently affecting the detected signal [32]. The impact of pressure on detection is not only observed between individuals but also occurs during a single measurement session [33]. Involuntary minor movements of the hand can lead to pressure fluctuations, which in turn cause variations in the detected signal [34].

To address the common challenges of existing SAF detection systems—namely, bulky size, high power consumption, and low integration level, which hinder their use in wearable and portable applications—this study designs and implements a miniaturized skin fluorescence detection system based on a CMOS sensor and a Bluetooth communication module. The system’s stability and fluorescence detection performance were evaluated. In practical tests, stable fluorescence signals were acquired across different exposure times and gain settings, demonstrating good linearity and SNR. The integration of a pressure sensor significantly reduced signal deviation caused by unstable contact, enhancing detection repeatability and reliability. All experiments were controlled via a mobile-phone application, with both control and detection signals transmitted via Bluetooth. The communication remained stable, and mobile operation was smooth.

## 2. Materials and Methods

**Figure 1:**
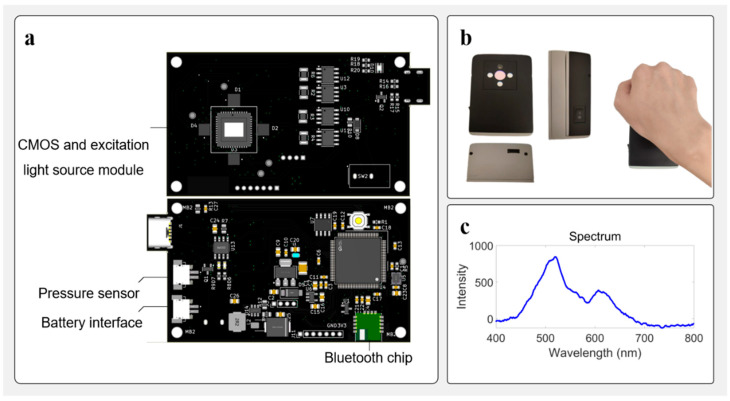
Overview of the autofluorescence detection system. (**a**) Front view of the printed circuit board (PCB) showing LEDs, the sensor, and associated analog circuitry responsible for detecting the emitted light from the sample. The back view of the same PCB, containing the microcontroller, power management circuit, LED driver, and communication interfaces. (**b**) Mechanical housing of the device and usage demonstration. The images show the front, side, and bottom views of the assembled device, along with a handheld operation scenario illustrating typical measurement posture. (**c**) The curve shows the emission fluorescent signal collected by a fiber optical spectrometer (USB2000, Ocean Optics).

This study aims to develop a low-power, portable skin autofluorescence (SAF) detection system optimized for wearable health monitoring and chronic disease risk screening. The system circuit and optical component design are shown in Figure 1a,b. The system is structured around a microcontroller, incorporating multi-wavelength excitation light, high-sensitivity optical and pressure sensors, and Bluetooth Low Energy (BLE) communication into a closed-loop acquisition and processing architecture.

### 2.1. STM32H750VB-Based Control Device

The STM32H750VB, a high-performance 32-bit microcontroller from STMicroelectronics, is built on the Arm^®^ Cortex^®^-M7 core and operates at up to 480 MHz [35]. It integrates a single-precision floating-point unit (FPU), digital signal processing (DSP) extensions, and the Thumb-2 instruction set, enabling efficient execution of computational tasks such as image filtering and numerical analysis for SAF signal processing [36]. With 1 MB of on-chip SRAM segmented into regions (e.g., DTCM, SRAM1, SRAM2), the system supports parallel data access and reduces latency. A 16 KB instruction/data cache further enhances memory efficiency, especially for external storage. The microcontroller includes three 16-bit ADCs (up to 3.6 Msps) for high-speed acquisition of analog signals such as pressure sensing and two 12-bit DACs for analog output control, useful in excitation light modulation and calibration tasks. A rich set of peripheral interfaces—eight UART/USART ports, six SPIs, and three I2C channels—facilitates integration with external modules. UART is used for real-time communication with the Bluetooth module, supporting wireless transmission of fluorescence and pressure data, while SPI and I2C interfaces handle memory devices, sensors, and control components. This modular, high-bandwidth architecture supports compact system design and scalability for wearable health monitoring applications.

### 2.2. Image Acquisition with AR0130 Sensor

Due to the low fluorescence quantum efficiency of autofluorescent substances in skin tissue, compared with specialized biological experimental dyes, the autofluorescence signal of the skin is relatively weak [37]. In the field of biomedical optics, APD, PMT, or photodiodes with preamplification function are usually used to detect bioluminescence signals [38]. In addition to the complex preamplifier circuit, the driving power supply is also an essential component for achieving APD, PMT, and photodiode detection [39]. Generally, APD and PMT need a high-voltage power supply to drive, which makes it difficult for these two detectors to be used in small equipment [40]. In the field of fluorescence imaging, highly sensitive CMOS/CCD is often used to obtain the spatial distribution information of fluorescence [41]. However, traditional CMOS/CCD usually requires processing by FPGA modules and data readout modules before being transmitted to a microcomputer [42]. For the field of wearable health monitoring, complex power drives, preamplifier circuits, and FPGA modules are not alternative approaches.

The detection of autofluorescence mainly focuses on the variation in light intensity in the temporal dimension [43]. Therefore, for the light intensity information collected by CMOS, the light intensity information can be obtained by integrating the gray values of all pixels. In this way, there is no need to save and transfer large-scale data. This system adopts the AR0130 CMOS sensor (1280 × 960 resolution, 8–12-bit output), optimized for low-light and high dynamic range environments. It interfaces directly with the DCMI (digital camera memory interface) module of the STM32H750VB microcontroller, enabling synchronized control of image capture through PCLK, HSYNC, and VSYNC signals. Real-time data transfer is achieved using DMA (direct memory access), which offloads data movement from the CPU, thereby reducing latency and system resource consumption. The captured image frames undergo grayscale averaging to extract fluorescence intensity values, which serve as the primary feature metric for SAF signal analysis.

To further ensure signal fidelity under weak fluorescence conditions, the system configures adjustable exposure time and analog gain parameters through software, enabling flexible adaptation to different skin types and excitation wavelengths. The AR0130 sensor’s high sensitivity in the near-UV and visible range enhances its capability to detect endogenous fluorophores such as NADH, AGEs, and FAD, even under short exposure times [44]. Additionally, the sensor’s low dark current and low read noise contribute to preserving a high signal-to-noise ratio, which is crucial for accurate fluorescence quantification in portable devices without active cooling [45].

In combination with the integrated SRAM and on-chip image processing capability of the STM32H750VB, the system achieves a compact and power-efficient fluorescence imaging solution. This approach avoids the need for external FPGAs or complex optical components, making it particularly suitable for wearable or mobile health monitoring platforms. The modular design also allows for future scalability, such as the addition of multi-frame fusion or motion compensation algorithms to further enhance stability and image quality under real-world usage conditions.

### 2.3. Multi-Wavelength Excitation and Spectral Matching

In terms of excitation light source configuration, the system integrates four high-brightness LEDs with central wavelengths of 365 nm, 395 nm, 415 nm, and 520 nm, respectively. The optical power for 365 nm, 395 nm, 415 nm, and 520 nm LEDs are 12.19 mw, 28.86 mw, 9.2 mw, and 4.1 mw, respectively. Among them, 365 nm, 395 nm, and 415 nm bands were used as multi-channel excitation sources, corresponding to the characteristic absorption peaks of endogenous fluorophores such as NADH (nicotinamide adenine dinucleotide) and AGEs (advanced glycation end products), respectively [46,47]. Herein, in order to obtain the spectral characteristics of skin fluorescence more comprehensively, we used a Y-type fiber optic probe, in which one fiber transmits 365 nm excitation light, and the other fiber collects fluorescence and transmits it to a fiber optical spectrometer (USB2000, Ocean Optics). The fluorescence spectrum collected by the fiber optic spectrometer is shown in Figure 1c. In this fluorescence spectrum, the fluorescence at 520 nm is stronger. Hence, we chose the 520 nm fluorescence signal as the detection signal in this work. The 520 nm LED is used for background calibration and comparative analysis of light source stability to provide a reference baseline for fluorescence signal quantification. The intensity of light emitted by the 520 nm LED is defined as d0, which can be obtained by a standard optical power meter. The detection light intensity signal d520 contributed by 520 nm light after diffuse transmission to the CMOS chip, d520/d0, represents the attenuation of the 520 nm signal caused by skin absorption and scattering. Since the positions of the four LEDs with different wavelengths are symmetrical, the ratio of the fluorescence signal excited by the three UV band LEDs to d520/d0 represents the true spontaneous fluorescence intensity of a skin surface. In this work, all fluorescence detection results are the ratio of original detection results to d520/d0.

**Figure 2:**
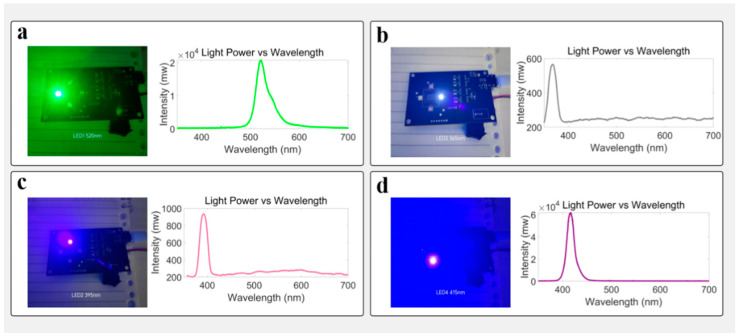
Overview of LED light sources and their corresponding spectral data. (**a**) Image of LED 4 (520 nm) and its corresponding spectral intensity curve. The visual appearance of the LED in operation is shown, alongside the intensity distribution captured by the spectrometer. (**b**) Image of LED 1 (365 nm) and its corresponding spectral intensity curve. The visual appearance of the LED is shown, alongside the spectral intensity data. (**c**) Image of LED 2 (395 nm) and its corresponding spectral intensity curve. The visual appearance of the LED is shown, along with the intensity distribution measured by the spectrometer. (**d**) Image of LED 3 (415 nm) and its corresponding spectral intensity curve. The visual appearance of the LED is displayed, along with its wavelength versus intensity data.

Meanwhile, the detection window is equipped with a bandpass filter with a central wavelength of 520 nm to suppress excitation light leakage and cover fluorescence emission regions. When using bandpass filters with other central wavelengths to collect fluorescence, fluorescence of different wavelengths will be corrected based on the quantum effect spectral curve of the sensor. The detection window is above the CMOS chip. During skin fluorescence monitoring, the skin clings to the detection window. The above settings enable the spontaneous fluorescence emitted from the skin to be detected by CMOS through a short optical path, improving the detection efficiency of spontaneous fluorescence. This configuration method of multi-band excitation and single-channel filtering detection helps to improve the signal-to-noise ratio and detection stability of the system under weak fluorescence conditions [48]. See Figure 2.

### 2.4. Wireless Communication and BLE Integration

For wireless control and efficient data transmission, the system incorporates a low-power Bluetooth module (BT24-T) on the PCB, which supports the Bluetooth Low Energy (BLE) protocol. This module features ultra-low standby current and a compact footprint, making it well-suited for wearable and mobile applications [49]. It communicates with the STM32H750VB microcontroller via UART, enabling real-time wireless transmission of key data such as fluorescence image features and pressure signals. Compared to Wi-Fi or traditional Bluetooth, BLE offers significantly lower power consumption, faster pairing, and more stable communication—an advantage in mobile health monitoring scenarios where battery life and portability are critical.

### 2.5. Pressure Sensing and Contact Calibration

To complement the wireless functionality and further enhance system robustness during data acquisition, a resistive pressure sensor module is integrated into the device. The analog output from this sensor is continuously sampled by the internal ADC of the STM32H750VB to monitor contact quality in real time. This pressure signal serves a dual purpose: it not only acts as a trigger to initiate image acquisition once a stable skin contact is confirmed—defined by exceeding a preset pressure threshold—but also serves as a correction parameter in subsequent signal analysis. Experimental observations suggest that mechanical pressure may alter local blood distribution, potentially affecting the absorption characteristics of both excitation and emission light. By incorporating pressure information into the processing pipeline, the system compensates for contact-induced signal fluctuations, ensuring more stable and reliable fluorescence readings. The resistive sensor design allows accurate pressure detection through voltage variation, thereby supporting real-time contact validation, automatic triggering, and post-processing correction mechanisms.

### 2.6. Closed-Loop Architecture and Data Integration

**Figure 3:**
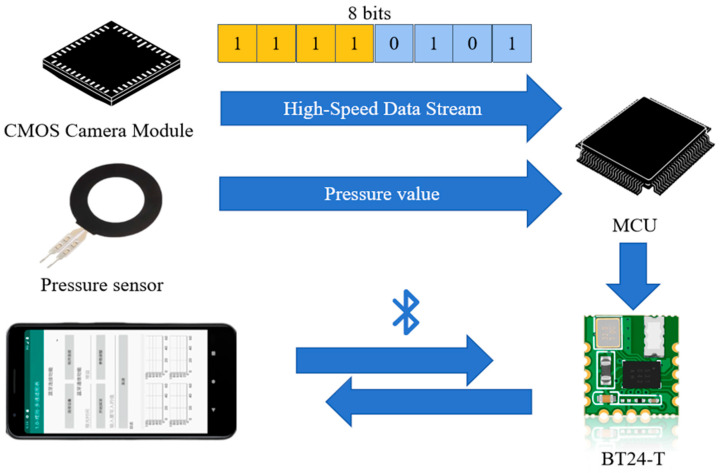
System architecture based on the STM32H750VB for SAF detection. incorporating image acquisition, pressure sensing, signal processing, and BLE wireless transmission for real-time mobile monitoring and interaction.

Building upon the integration of these components, the complete SAF detection architecture is constructed around the STM32H750VB microcontroller, achieving a compact, closed-loop system that encompasses image acquisition, signal processing, and wireless communication. Fluorescence signals are captured by the AR0130 CMOS sensor and streamed through the DCMI interface, with synchronization via PCLK, HSYNC, and VSYNC signals enabling real-time image scanning. To improve data throughput and reduce CPU load, data are transferred to RAM using DMA. The system computes the average grayscale intensity of the captured image to quantify the SAF signal. Simultaneously, the analog signal from the pressure sensor is digitized for contact validation and fluorescence correction. Both the grayscale and pressure data are transmitted via UART to the Bluetooth module and sent over BLE to a mobile terminal. The Android application supports real-time data visualization, local storage, and remote parameter configuration (e.g., exposure time, gain), forming a user-interactive feedback loop suitable for dynamic monitoring in wearable scenarios. See Figure 3.

## 3. Results

### 3.1. Skin Autofluorescence Signal Acquisition and Processing

The portable SAF detection system uses excitation wavelengths of 365 nm, 395 nm, and 415 nm, with a detection channel centered at 520 nm, coupled with an external bandpass filter. The detection module is based on the AR0130 CMOS sensor, which is interfaced via a DVP parallel port with the STM32H750VB microcontroller. The measurement site was the central region of the palm. The system autonomously controls the excitation light, captures the signal, and reads out data. For each subject, six frames at a resolution of 1280 × 960 were acquired. The system calculates the average pixel intensity across each frame, serving as the autofluorescence intensity signal (on a scale of 0–255). Regarding the suppression of photobleaching, first of all, the power of our excitation light is low (10–30 mw), and the exposure time of CMOS is also short (100 ms). The light source will automatically turn off after being turned on for 100 ms, which can effectively suppress photobleaching. In addition, the detection of skin fluorescence is different from the detection of pulse wave PPG signals and does not need to be turned on for a long time. According to the medical application of AGEs, skin fluorescence detection once a day can provide sufficient medical information. Therefore, the light dose of the skin is relatively small, and photobleaching can be ignored.

### 3.2. Light Avoidance Test

**Figure 4:**
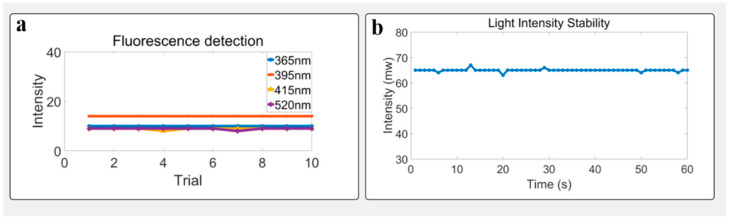
System performance and stability analysis: (**a**) System background response under full shielding of both the excitation source and detection area using a black metal sheet. Across all excitation wavelengths (365 nm, 395 nm, 415 nm, and 520 nm), the background grayscale values remained consistently low with minimal variation, demonstrating the system’s effective suppression of ambient and stray light. (**b**) Light intensity stability over a 60 s period using the 520 nm LED source. The plot shows minimal fluctuations in light intensity, confirming the stability of the light source during this short time period.

To assess the system’s baseline response under complete darkness, both the excitation light source and the detection area were fully covered using a black metal sheet to block all incident light. This configuration simulates a dark-field environment and is designed to evaluate the system’s intrinsic noise characteristics and the dark current behavior of the image sensor. Measurements were conducted under four nominal excitation wavelengths (365 nm, 395 nm, 415 nm, and 520 nm). The average grayscale value of each captured image was recorded. Results showed that, in the absence of excitation light, grayscale values across all wavelengths remained below 20 with fluctuations within ±1 unit, indicating low baseline noise and excellent stability in dark-state operation. These findings confirm that the system maintains minimal background interference under fully shielded conditions, providing a reliable foundation for detecting weak fluorescence signals in practical applications. In the subsequent fluorescence detection experiments, the fluorescence data we obtained were all subtracted from the background dark noise. See Figure 4.

### 3.3. Parameter Tuning and Fluorescence Response

**Figure 5:**
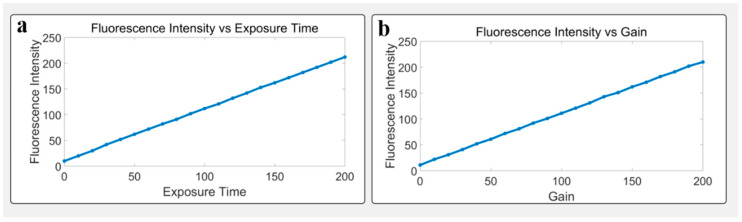
Linear fluorescence response to system parameters: (**a**) exposure time and (**b**) gain, showing strong linearity.

This system can adjust the exposure time and gain through Bluetooth commands. This function is very important for the detection of individuals with different spontaneous fluorescence intensities. To examine how the system responds to different exposure and gain settings, average grayscale values were measured across various configurations. Results revealed strong linear relationships between fluorescence intensity and both exposure time (0–200 ms) and gain (0–200 dB), with R^2^ > 0.98, indicating robust linear responsiveness suitable for automated control and quantification. This experiment shows that under the exposure time of 200 microseconds, the system can obtain fluorescence signals with large amplitude, which is very beneficial for rapid monitoring and is also very friendly to the detection accuracy and user experience. See Figure 5.

### 3.4. Stability Under Static Conditions

**Figure 6:**
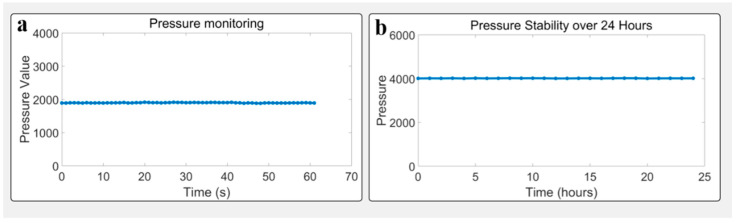
Pressure monitoring during SAF acquisition: (**a**) stable contact pressure; (**b**) pressure stability over a 24 h period with a 200 g sample.

To verify the system stability of pressure monitoring under constant illumination and background, repeated measurements (*n* = 30) were conducted on a fixed subject. Before the hand is placed on the instrument, the pressure value is about 2000 due to the weight of the instrument itself. After the hand is placed on the instrument, the pressure value increases to about 3000. Pressure value variation remained within ±2.5%, and pressure fluctuation within ±3.0%. Time-series plots showed no drift or sudden shifts, confirming the system’s short-term stability and repeatability. See Figure 6.

### 3.5. Influence of Age and Body Weight on Fluorescence

A total of five healthy volunteers were recruited for this study, including three males and two females, aged between 3 and 60 years. All participants were in good health prior to testing, with no history of chronic diseases, skin disorders, or recent medication. The tested skin area was free from visible injuries, scars, or abnormal pigmentation. During this study, no participants underwent dietary, exercise, or hypoglycemic interventions, nor were they receiving treatments for any relevant conditions. For the standard medical instrument AGEs Reader, based on the literature data, it mainly uses ultraviolet light to illuminate the skin, divides the fluorescence by the excitation light, and uses visible light to calibrate the effect of skin color. Our work refers to the method of the standard medical instrument AGEs Reader to achieve miniaturized skin fluorescence detection. The test results have a certain correlation with age.

AGEs are formed in the non-enzymatic reaction (Maillard reaction) of reducing sugars with amino residues on proteins, lipids, or nucleic acids. Once formed, AGEs are extremely difficult to degrade, especially long-term accumulation on long-lived proteins (such as collagen), and gradually accumulate with age. Therefore, this work uses the method of skin fluorescence detection to analyze the relationship between AGEs autofluorescence and aging. To assess the correlation between SAF and physiological factors, subjects of varying age and weight were tested under fixed conditions (exposure: 100 ms; gain: 10 dB) using excitation at 365, 395, and 415 nm. All images were taken from the same palm region, avoiding pigmentation, vessels, and scars. Each subject contributed three rounds of detection, with mean grayscale as the fluorescence indicator. Pressure values were also recorded to ensure consistency.

The accumulation of AGEs in the skin is a long process, and it remains basically unchanged in short-term measurements. For example, we used AGEs Reader to continuously test a 30-year-old volunteer for 7 days, and the test results were 1.4, 1.6, 1.6, 1.6, 1.5, 1.4, and 1.4. The values here were generated by AGEs Reader, and the unit was also customized by AGEs Reader. Using the system of this work, under 415 nm excitation, the 7-day test results were 18, 17, 17, 19, 19, 17, and 28, which were relatively stable.

At 395 nm excitation, fluorescence intensity increased nearly linearly with age from 3 to 60 years (R^2^ = 0.95). At 365 nm, the signal rose significantly after the age of 30, peaking around 40 and then slightly declining. At 415 nm, fluorescence remained relatively stable with a peak near 40. These findings suggest 395 nm is most sensitive to age-related metabolic changes, potentially linked to AGEs accumulation and collagen structure. See Table 1.

In terms of body weight, subjects ranging from 14 to 70 kg showed a positive linear correlation between weight and fluorescence intensity under 395 nm (R^2^ = 0.92). Similar but smaller trends were observed under 365 and 415 nm. In this optical system, the 415 nm wavelength is comparatively longer, resulting in lower excitation efficiency; thus, the fluorescence generated under 415 nm irradiation is relatively weak. Additionally, as skin melanin exhibits stronger absorption of shorter-wavelength light, the fluorescence excited by 365 nm light is also weak. See Table 2.

**Figure 7:**
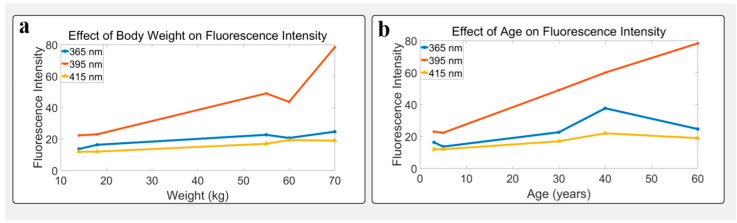
Influence of physiological parameters on skin autofluorescence intensity: (**a**) fluorescence intensity trend with age; (**b**) fluorescence intensity trend with body weight. Under 395 nm excitation, signals show a clear positive correlation with both age and weight, indicating strong sensitivity to metabolic accumulation such as AGEs.

Overall, the 365, 395 nm, and 415 excitation wavelengths can stimulate the fluorescence of skin. It effectively differentiated fluorescence characteristics among individuals of varying ages and body weights. Combined with the pressure sensor’s synchronized correction function, the developed system shows strong feasibility for quantitatively analyzing skin metabolic activity and physiological states. It holds promising potential for future applications in personalized health monitoring and chronic disease management. See Figure 7.

## 4. Discussion

This work focuses on the integration of high-sensitivity sensors into miniaturized control circuits and their application in skin fluorescence detection. First of all, the introduction of CMOS ensures the advantages of high integration, strong controllability, and high sensitivity of the sensor. In addition, CMOS has the ability to collect spatial information and can detect extremely weak signals through long exposure time. However, the hardware interface of CMOS sensors is complex and usually requires the use of general-purpose computer chips for control. For portable devices, reducing power consumption is also a very important indicator. This work uses the STM32 microcontroller and tailors the functions of CMOS in a targeted manner, retaining the unnecessary exposure gain control function, which not only realizes the control of CMOS chips using low-power microcontrollers but also ensures that the basic detection capabilities are not affected.

According to the experimental results under a dark background, this system has low and stable dark noise, which provides favorable conditions for the follow-up weak autofluorescence detection. To address inter-individual variation in optical properties, particularly those arising from differences in skin pigmentation, thickness, and melanin content, the system incorporates a 520 nm LED as a built-in reflectance calibration reference. Following fluorescence acquisition, the system sequentially illuminates the same skin site with 520 nm light, and the resulting diffuse reflectance signal is captured under identical optical and geometric conditions. This enables the computation of a fluorescence-to-reflectance ratio, effectively normalizing intensity variations due to skin-dependent light scattering and absorption. Given the short exposure duration (100 ms), spatial displacement caused by involuntary movement is negligible, ensuring that both fluorescence and reflectance originate from a spatially co-registered tissue volume, thereby enhancing normalization fidelity.

In this work, a 520 nm bandpass filter is selected to extract the autofluorescence of relevant wavebands, which takes into account the crosstalk of spontaneous fluorescence. Collagen is the main contributing element to the SAF. The autofluorescence of collagen units is mainly in the 400–500 nm band. It is mentioned in the literature [50] that with the process of glycosylation, collagen would gradually be converted into glycosylated products, and the spectrum of its spontaneous fluorescence would be red shifted. Therefore, in this paper, we choose to monitor the spontaneous fluorescence intensity at 520 nm. Compared to the 400 nm–500 nm band, the fluorescence in the 520 nm band comes more from AGEs, which can better analyze AGEs.

Among the excitation sources tested (365, 395, 415 nm), the 395 nm wavelength demonstrated the highest signal responsivity and sensitivity. Part of the reason is that among the LEDs we have chosen, the 395 nm LED has the highest luminous efficiency, particularly to variations associated with age and metabolic indicators such as body mass index (BMI). This is consistent with the known excitation spectrum of advanced glycation end-products (AGEs), a class of autofluorescent molecules that accumulate in long-lived dermal proteins due to oxidative stress and chronic hyperglycemia. AGEs are increasingly recognized as early, noninvasive biomarkers for type 2 diabetes, cardiovascular disease, and systemic inflammation. The observed correlation between SAF intensity under 395 nm excitation and subject-specific metabolic features underscores the potential of this system in longitudinal health risk profiling.

Although this study is limited in cohort size and wavelength granularity, the results validate the sensitivity and robustness of the system and highlight its potential as a scalable platform for continuous metabolic monitoring and early disease detection in decentralized healthcare settings.

## 5. Conclusions

This study presents a miniaturized skin autofluorescence (SAF) detection system with an emphasis on integrated hardware design, low-power operation, and real-time multisource data processing. Unlike traditional platforms reliant on high-intensity excitation sources and complex optical assemblies, the proposed solution demonstrates the feasibility of acquiring biologically relevant optical signals under constrained power and form-factor conditions—key features for wearable and point-of-care applications.

This mobile and wireless SAF detection system achieves a functional balance between miniaturization and high-quality signal acquisition. It demonstrates technical feasibility in acquiring physiological signals from the skin in dynamic or minimally supervised environments.

The system’s architecture supports modular upgrades. The onboard STM32H750VB microcontroller, with an embedded DMA controller and DCMI interface, enables direct high-throughput acquisition from the AR0130 CMOS sensor, reducing CPU burden and supporting real-time grayscale analysis. Grayscale intensity and pressure data are jointly transmitted via BLE to a mobile terminal for visualization and parameter control. The architecture is fully compatible with AI integration pipelines. In future iterations, machine learning algorithms (e.g., CNNs or XGBoost) can be trained on fluorescence–reflectance–pressure feature vectors to classify risk levels, predict disease progression, or stratify patient populations.

In addition to spectral optimization, the system integrates a resistive pressure sensor for real-time assessment of contact quality. Variations in contact pressure alter local dermal perfusion and hemoglobin concentration, both of which are significant absorbers at UV–visible wavelengths. The system uses pressure-derived analog signals to dynamically determine when sufficient contact is established (exceeding a predefined threshold) and to adjust fluorescence signal interpretation accordingly. This dual-use of the pressure signal—both as a trigger and as a normalization variable—enables improved measurement repeatability in self-administered or motion-prone scenarios.

The system exhibits a stable optical response across multiple excitation wavelengths within a 100 ms exposure time. By integrating a real-time pressure feedback mechanism, the system enhances the consistency and reliability of fluorescence readings, providing a robust basis for further biomedical applications. Using this system, SAF intensity of individuals of different ages and weights can be monitored. Its strength trend is basically consistent with that described in the literature.

The current detection window is equipped with a 520 nm bandpass filter, which can only detect autofluorescence signals in the 520 nm band. In future work, we will introduce multispectral technology to increase the number of detection bands and better separate the signal crosstalk generated by different autofluorescence agents.

In future work, we will improve the detection rate of the system, obtain the dynamic characteristics of spontaneous fluorescence, especially the time variation law of spontaneous fluorescence under pulse wave modulation, and overcome the changes in absorption coefficient of biological tissues caused by changes in blood volume caused by cardiac pulsation.

Overall, the proposed system shows promising potential for transitioning from point-of-care diagnostics to continuous health monitoring. It may serve as a foundation for future integration into home-use medical devices, personalized metabolic risk evaluation, and intelligent digital health platforms.

The portable sensors developed in this work have the potential to become wearable sensors in the future, but there are still two major challenges that need to be overcome. First, there is the challenge of miniaturization of devices. To ensure the stability of the system, this work currently mainly uses components with a package size of 0402 (about 1.0 mm long and about 0.5 mm wide) and above. In the future development of wearable devices, it is necessary to consider using components with a smaller package size (such as 0201). Small components will make design and production difficult and will also affect the stability of the system. Second, skin fluorescence detection involves both the emission of excitation light and the detection of signals. Because skin is a highly scattering tissue, the sensor and the light source need to be as close as possible to ensure an efficient autofluorescence detection rate. However, when the light source and the sensor are too close, there will be interference between the electrical characteristics of the two components, which requires special consideration in miniaturized design.

This miniaturized system integrates the necessary LEDs and light detectors. In addition to the autofluorescence detection of AGEs, it can also be used in bilirubin fluorescence monitoring. In addition, combined with pulse wave monitoring technology, the system can also be used to monitor heart rate and blood oxygen saturation, ultimately realizing a multi-parameter portable or wearable health monitoring tool.

## Figures and Tables

**Table 1:** Fluorescence intensity at different ages (mean grayscale values).

Age (yrs)	Sex	365 nm^−1^	365 nm^−2^	365 nm^−3^	365 nm-Mean	365 nm-Std	395 nm^−1^	395 nm^−2^	395 nm^−3^	395 nm-Mean	395 nm-Std	415 nm^−1^	415 nm^−2^	415 nm^−3^	415 nm-Mean	415 nm-Std
3	M	24	13	12	16.33	6.51	23	19	27	23.00	4.00	11	11	14	12.00	1.73
5	F	14	14	13	13.67	0.58	19	22	26	22.33	3.51	11	11	14	12.00	1.73
30	F	24	17	27	22.67	5.13	49	43	55	49.00	6.00	18	14	19	17.00	2.65
40	M	39	27	47	37.67	10.07	72	61	47	60.00	12.75	27	21	18	22.00	4.58
60	M	23	22	29	24.67	3.79	82	82	71	78.33	6.35	19	22	16	19.00	3.00

**Table 2:** Fluorescence intensity at different body weights (mean grayscale values).

Weight (kg)	Sex	365 nm^−1^	365 nm^−2^	365 nm^−3^	365 nm-Mean	365 nm-Std	395 nm^−1^	395 nm^−2^	395 nm^−3^	395 nm-Mean	395 nm-Std	415 nm^−1^	415 nm^−2^	415 nm^−3^	415 nm-Mean	415 nm-Std
14	F	14	14	13	13.67	0.58	19	22	26	22.33	3.51	11	11	14	12.00	1.73
18	M	24	13	12	16.33	6.51	23	19	27	23.00	4.00	11	11	14	12.00	1.73
55	F	24	17	27	22.67	5.13	49	43	55	49.00	6.00	18	14	19	17.00	2.65
60	M	21	21	20	20.67	0.58	42	46	43	43.67	2.08	19	20	19	19.33	0.58
70	M	23	22	29	24.67	3.79	82	82	71	78.33	6.35	19	22	16	19.00	3.00

## Data Availability

Data available upon request from the authors.
